# RISK FACTORS FOR SEVERE POSTOPERATIVE COMPLICATIONS AFTER GASTRECTOMY FOR GASTRIC AND ESOPHAGOGASTRIC JUNCTION CANCERS

**DOI:** 10.1590/0102-672020190001e1473

**Published:** 2019-12-20

**Authors:** Enrique NORERO, Jose Luis QUEZADA, Jaime CERDA, Marco CERONI, Cristian MARTINEZ, Ricardo MEJÍA, Rodrigo MUÑOZ, Fernando ARAOS, Paulina GONZÁLEZ, Alfonso DÍAZ

**Affiliations:** 1 Hospital Dr. Sotero del Rio, Esophagogastric Surgery Unit, Digestive Surgery Department, Pontificia Universidad Catolica de Chile, Chile; 2 Epidemiology Department, Department of Public Health, Faculty of Medicine, Pontificia Universidad Catolica de Chile, Chile.

**Keywords:** Stomach neoplasms, Gastrectomy, Risk factors, Morbidity, Adenocarcinoma, Neoplasias gástricas, Gastrectomia, Fatores de risco, Morbidade, Adenocarcinoma

## Abstract

**Background:**

Gastrectomy is the main treatment for gastric and Siewert type II-III esophagogastric junction (EGJ) cancer. This surgery is associated with significant morbidity. Total morbidity rates vary across different studies and few have evaluated postoperative morbidity according to complication severity.

**Aim:**

To identify the predictors of severe postoperative morbidity.

**Methods:**

This was a retrospective cohort study from a prospective database. We included patients treated with gastrectomy for gastric or EGJ cancers between January 2012 and December 2016 at a single center. Severe morbidity was defined as Clavien-Dindo score ≥3. A multivariate analysis was performed to identify predictors of severe morbidity.

**Results:**

Two hundred and eighty-nine gastrectomies were performed (67% males, median age: 65 years). Tumor location was EGJ in 14%, upper third of the stomach in 30%, middle third in 26%, and lower third in 28%. In 196 (67%), a total gastrectomy was performed with a D2 lymph node dissection in 85%. Two hundred and eleven patients (79%) underwent an open gastrectomy. T status was T1 in 23% and T3/T4 in 68%. Postoperative mortality was 2.4% and morbidity rate was 41%. Severe morbidity was 11% and was mainly represented by esophagojejunostomy leak (2.4%), duodenal stump leak (2.1%), and respiratory complications (2%). On multivariate analysis, EGJ location and T3/T4 tumors were associated with a higher rate of severe postoperative morbidity.

**Conclusion:**

Severe postoperative morbidity after gastrectomy was 11%. Esophagogastric junction tumor location and T3/T4 status are risk factors for severe postoperative morbidity.

## RESUMO

**Raciona:**

l: A gastrectomia é o tratamento principal para o câncer de junção esofagogástrica (EGJ) e Siewert tipo II-III. Ela está associada à morbidade significativa. As taxas de morbidade total variam entre os diferentes estudos e poucos avaliaram a morbidade pós-operatória de acordo com a gravidade da complicação.

**Objetivo:**

Identificar os preditores de morbidade pós-operatória grave.

**Métodos:**

Este foi um estudo de coorte retrospectivo de um banco de dados prospectivo. Foram incluídos pacientes tratados com gastrectomia para câncer gástrico ou EGJ em um único centro. A morbidade severa foi definida como escore de Clavien-Dindo ≥3. Análise multivariada foi realizada para identificar preditores de morbidade grave.

**Resultados:**

Duzentos e oitenta e nove gastrectomias foram realizadas (67% homens, mediana de idade: 65 anos). A localização do tumor foi EGJ em 14%, o terço superior do estômago em 30%, o terço médio em 26% e o terço inferior em 28%. Em 196 (67%), foi realizada gastrectomia total com dissecção de linfonodos D2 em 85%. Duzentos e onze pacientes (79%) foram submetidos à gastrectomia aberta. O estado T foi T1 em 23% e T3/T4 em 68%. A mortalidade pós-operatória foi de 2,4% e a taxa de morbidade foi de 41%. A morbidade severa foi de 11% e foi representada principalmente por fístula esofagojejunal (2,4%), fístula duodenal (2,1%) e complicações respiratórias (2%). Na análise multivariada, a localização do EGJ e os tumores T3/T4 foram associados com maior morbidade pós-operatória grave.

**Conclusão:**

Morbidade pós-operatória severa após gastrectomia foi de 11%. A localização do tumor na junção esofagogástrica e o estado T3/T4 são fatores de risco para a morbidade pós-operatória grave.

## INTRODUCTION

Gastric cancer (GC) is the fifth most common cancer^9^, with more than 900,000 new cases every year, and the third leading cause of cancer-related death worldwide^29^. Surgery and adjuvant treatment are the main treatment modalities for GC. The gastrectomy is the approach universally agreed upon for gastric and Siewert type III esophagogastric junction (EGJ) cancer^15^ ^,^ ^24^. Although controversies exists respect the treatment for Siewert II tumors, the extended total gastrectomy also appear as an appropriate surgical option^5^ ^,^ ^10^. Postoperative morbidity rates after gastrectomy vary across different studies, but total morbidity is more than 20-30% in most studies^3,12,13,18,22,23,^. In a previous study by our group, morbidity was present in 31% of 1066 gastrectomies^21^.

Data on postoperative morbidity predictors are heterogeneous. Patient (age, comorbidity, body mass index, serum albumin), tumor (local invasion and location), and surgery (open approach, total gastrectomy, lymph node dissection, and multi-organ resection) variables are described as potential factors for higher morbidity^3^ ^,^ ^22^ ^,^ ^23^.

In our previous study, we did not have data on complication severity because this type of score did not exist at the beginning of that study^21^. In the past decade, complication severity has gained great importance, and use of the Clavien-Dindo classification has been widely adopted^8^. However, only a few studies have evaluated postoperative morbidity predictors according to complication severity for gastrectomy^13^ ^,^ ^18^.

The aim of this study was to identify predictors of severe postoperative morbidity after gastrectomy for gastric (GC) and esophagogastric junction (EGJ) cancer.

## METHODS

The local ethics committee approved this study. Informed consent of patients was waived because of the retrospective nature. This study was registered in ClinicalTrial.gov NCT03909997.

This was a retrospective cohort, including data from a prospective, institutional, single-center database. The database collected patients’ demographics, tumor and surgery characteristics, and postoperative morbidity. All consecutive patients treated with a gastrectomy for GC or EGJ cancer between January 2012 and December 2016 were included. Only patients with stomach or EGJ adenocarcinoma were selected, and patients with other histology were excluded.

### Preoperative assessment

The preoperative assessment consisted of upper gastrointestinal endoscopy, biopsy, complete blood count, liver function tests, electrocardiogram, and nutritional evaluation. Patients with diabetic, coronary heart disease and COPD were assessed additionally with a glycated hemoglobin test, echocardiogram and spirometry respectively. Preoperative imaging was a thorax-abdomen-pelvis computed tomography (CT) scan.

### Operative procedure

Epidural analgesia was routinely employed in open surgery. Depending on the tumor’s location, a total or subtotal gastrectomy was indicated. Surgery included omentectomy with bursectomy and D2 lymph node dissection, according to the Japanese classifications in patients with curative gastrectomy^25^. Multi-organ resection, including spleen, pancreas, colon, and liver, was performed in cases of direct tumor invasion. Partial distal esophagectomy with a transhiatal approach and mediastinal anastomosis was employed for Siewert types II and III cancers, with frozen section intraoperative biopsy to confirm an R0 resection. Partial distal esophagectomy was considered a multi-organ resection when more than 2 cm of the esophagus was resected. Routine cholecystectomy was performed in curative cases and was not considered a multi-organ resection. A reconstruction, using Roux-en-Y, was performed after a total gastrectomy; Roux-en-Y or Billroth II was used for subtotal gastrectomy. Esophagojejunal anastomosis was performed with a circular stapler and a second layer of running monofilament suture. One or two prophylactic drains were used routinely^21^. A laparoscopic approach was employed in patients with clinical early GC who were not candidates for endoscopic resection and patients with advanced GC without clinical invasion of adjacent structures and with lymph node metastases only in the perigastric area^20^.

### Postoperative management

Patients started early respiratory and physical therapy the day following surgery. An oral contrast study was performed on postoperative day 5-7 for total gastrectomy; after this, the patient started an oral diet and prophylactic drains were removed.

Esophagogastric junction (EGJ) cancer was classified according to Siewert classification^27^ Only EGJ cancer Siewert types II and III were included, type I were excluded. Patients were staged using TNM Classification of Malignant Tumors, 7^th^ edition^19^.

### Complication assessment

The primary outcome was severe 30-day or in-hospital morbidity, which was defined as a Clavien-Dindo score ≥3^8^. The complication data were prospectively collected by each attending surgeon and together at a monthly conference. Complications detected upon readmission were also included.

Postoperative bleeding was defined as any blood loss through abdominal drains or at reoperation. We considered an esophagojejunostomy leak as the appearance of contrast outside the anastomosis, using an oral contrast or CT scan, or by direct evaluation at reoperation. We considered a duodenal stump leak as the discharge of bile-containing liquid in drains or by direct evaluation at reoperation. A pancreatic fistula was considered as a drain output of any volume on or after postoperative day 3 with an amylase greater than three times the serum level. Intra-abdominal abscess was defined as septic fluid in the abdominal cavity on CT causing systemic inflammatory response syndrome. Postoperative pancreatitis was diagnosed with elevated levels of pancreatic enzymes and/or imaging finding^2^.

### Sample size

Using previously published data and given a 0.05 alpha level, a percentage of unexposed outcome of 10.8%, and OR 4.28, a sample size of 116 patients would yield at least 80% statistical power.

### Statistical analysis

Continuous variables were described by means and standard deviations or medians and interquartile range. Categorical variables were described with frequencies and percentage. The following factors were analyzed: age, gender, comorbidity, American Society of Anesthesiologists (ASA) physical status, tobacco and alcohol consumption, body mass index (BMI), hematocrit, serum albumin level, tumor location, the use of preoperative chemotherapy, laparoscopic or open surgery, total or subtotal gastrectomy, duodenal stump closure, multi-organ resection, lymphadenectomy, reconstruction method, T status, lymph node metastasis, and resection margin. We used a cut-off point of 65 years for statistical analysis^17^. The T stage was grouped by T1/T2 and T3/T4 for analysis. Univariate and multivariate analyses were performed to identify predictors of severe postoperative morbidity. All variables associated with severe morbidity with p<.05 in the univariate analysis were subsequently entered into a Cox multivariate regression model with backward elimination. Significance was set at two-sided p <.05. All analyses were performed using the statistical SPSS IBM Statistics software program, version 22.

## RESULTS

Two hundred and eighty-nine gastrectomies were performed, 195 (67.5%) of whom were male, with a median age of 65 years (+/- 11). The median BMI was 24.4 (21.8-26.9), and median albumin was 4.1 gr/dl (3.6-4.4). The patients’ characteristics are summarized in Table 1. Eighty-three percent of patients had at least one comorbidity; the majority had ASA II (57%). Eight patients (2.8%) received neoadjuvant chemotherapy. The tumor was located in the EGJ in 14% of patients and in the stomach in 85%.

**TABLE 1:** Patients characteristics, tumor location, procedure data and tumor pathology (n=289)

Patients	n=289	(%)
Age, median (sd)	65	(11)
Male	195	(67.5)
Comorbidity	235	(83)
Arterial hypertension	124	(42.9)
Diabetes	42	(14.5)
Coronary heart disease	35	(12.4)
Chronic liver disease	12	(4.2)
ASA score		
I	88	(31.2)
II	161	(57.1)
III	33	(11.7)
Tabacco consumption	62	(22)
Alcohol consumption	18	(6.4)
Body mass index		
< 18.5	13	(4.5)
18.5 - 24.9	157	(54.3)
25 - 29.9	86	(29.8)
≥ 30	33	(11.4)
Hematocrit <30%	32	(11)
Albumin <3.0 g/dl	14	(4.9)
Tumor location		
Esophagogastric junction	42	(14.5)
Siewert II	14	(4.8)
Siewert III	28	(9.7)
Stomach	247	(85.4)
Upper third	89	(30.8)
Middle third	76	(26.3)
Lower third	82	(28.4)
Procedures		
Gastrectomy		
Open gastrectomy	231	(79.9)
Laparoscopic gastrectomy	58	(20.1)
Gastrectomy		
Total gastrectomy	196	(67.8)
Subtotal distal gastrectomy	93	(32.2)
Duodenal closure		
Hand-sewn	166	(42.5)
Mechanical	123	(57.5)
Multiorgan resection	69	(23.9)
Distal esophagectomy	37	(12.8)
Splenectomy	25	(8.6)
Pancreatectomy	18	(6.2)
Colectomy	6	(2)
Diaphragm resection	5	(1.7)
Liver resection	4	(1.4)
Duodenal resection	4	(1.4)
Total esophagectomy	2	(0.6)
Adrenalectomy	1	(0.3)
Lymph node dissection		
D2	245	(84.7)
D1 or D1+	44	(15.2)
Reconstruction route		
Retrocolic	183	(72.6)
Tumor pathology		
T Status		
T1	68	(23.5)
T2	24	(8.3)
T3	65	(22.5)
T4	132	(45.7)
Lymph node status		
N (-)	107	(37)
N (+)	182	(63)
Resection Margin		
R0	249	(86.2)
R1	1	(0.3)
R2	39	(13.5)

The open approach was employed in 231 (79.9%) patients. Total gastrectomy was performed in 196 patients (67.8%). It was necessary to perform a multi-organ resection in 69 cases (23.9%). Distal esophagectomy (12.8%), splenectomy (8.6%), and pancreatectomy (6.2%) were the most commonly resected organs. The majority of patients underwent a D2 dissection (84%). The alimentary tract reconstruction was a Roux-en-Y in 260 (89.9%) patients, the great majority with a retrocolic reconstruction (Table 1).

A complete resection (R0) was performed in 249 (86.2%). All 39 (13.5%) patients with an R2 resection had distant metastases. Twenty-three percent of patients had early GC (T1), and 68% had T3/T4 status. Lymph node metastases were diagnosed in 63%. The median number of resected lymph nodes was 34 (25-47), 76.8% of patients had a lymph node count of 25 or more lymph nodes, and 272 (94.1%) had 15 or more nodes resected.

**FIGURE 1:**
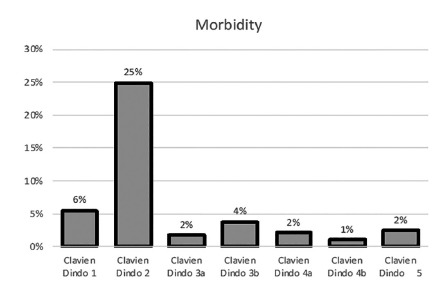
Postoperative morbidity according to Clavien-Dindo classification

Postoperative morbidity was present in 41.5% of patients. An intra-abdominal complication occurred in 26.3%, wound-abdominal wall complications were present in 4.8% of patients, and 19% had medical complications. Postoperative mortality was 2.4% (n=7). Severe morbidity occurred in 11% (n=32) (Figure 1). Patients with severe postoperative morbidity had a significantly longer postoperative stay (26±19 vs. 11±8; p<0.05). Esophagojejunal anastomosis leak (2.4%), duodenal stump leak (1.7%), and respiratory complication (2%) were the main severe complications (Table 2).

**TABLE 2:** Postoperative global and severe complications after gastrectomy

Postoperative morbidity	Severe	Global
	n (%)	n (%)
Intra-abdominal complication	22 (7.6)	76 (26.3)
Esophagojejunal anastomosis leak*	7 (2.4)	12 (4.2)
Duodenal stump leak	5 (1.7)	8 (2.8)
Pancreatic fistula	4 (1.4)	19 (6.6)
Intestinal injury	2 (0.7)	2 (0.7)
Jejuno-jejunal anastomosis leak†	1 (0.4)	1 (0.4)
Intra-abdominal bleeding	1 (0.4)	6 (2.1)
Intra-abdominal collection/abscess	1 (0.3)	25 (8.7)
Jejunostomy site obstruction	1 (0.3)	1 (0.3)
Ascites	0 (0)	5 (1.7)
Pancreatitis	0 (0)	4 (1.4)
Prolonged postoperative ileus	0 (0)	4 (1.4)
Wound - abdominal wall complication	0 (0)	14 (4.8)
Abdominal wall dehiscence	0 (0)	7 (2.4)
Surgical site infection	0 (0)	6 (2.1)
Seroma	0 (0)	2 (0.7)
Medical complication	10 (3.5)	55 (19)
Respiratory	6 (2)	17 (5.9)
Pneumonia	3 (1)	9 (3.1)
Pleural effusion	2 (0.7)	5 (1.7)
Atelectasis	1 (0.3)	3 (1)
Cardiovascular	4 (1.4)	16 (5.5)
Arrhythmia	2 (0.7)	4 (1.4)
Pericardial effusion	1 (0.3)	1 (0.3)
Pulmonary embolism	1 (0.3)	1 (0.3)
Deep venous thrombosis	0 (0)	10 (3.5)
Renal	0 (0)	13 (4.5)
Urinary tract infection	0 (0)	7 (2.4)
Acute renal failure	0 (0)	6 (2.1)
Other Infectious	0 (0)	17 (5.9)
*Clostridium difficile* infection	0 (0)	12 (4.2)
Central venous catheter sepsis	0 (0)	5 (1.7)
Neurologic	0 (0)	3 (1)
Delirium	0 (0)	3 (1)
Total	32 (11)	120 (41.5)

The esophagojejunal anastomosis leak rate was 4.2%. Five cases (1.7%) of esophagojejunal anastomosis leak were managed with nothing by mouth, antibiotics, and prophylactic drains, corresponding to a Clavien-Dindo score of II. Severe esophagojejunal anastomosis leak was diagnosed in seven cases (2.4%). Three patients underwent a reoperation due to clinical deterioration or abdominal abscess and later recovered. In one case of conservative treatment, the patient developed anastomotic stenosis and required an endoscopic dilation as the only intervention. Three patients who died due to esophagojejunal fistula had mediastinal and uni/bilateral pleural contamination; two of these patients underwent a reoperation, and one was treated conservatively, dying shortly after, due to multiple organ failure. Esophagojejunal anastomosis leak rate was 11.9% in patients with an EGJ tumor location.

Duodenal stump leak developed in 2.8% of patients. Three cases (1%) of duodenal stump fistula were managed with antibiotics and prophylactic drains or spontaneous drainage through the abdominal wound, corresponding to a Clavien-Dindo score of II. Five patients (1.7%) underwent reoperation due to duodenal stump leak, and in one of them a duodenostomy was performed. One patient underwent reoperation because the duodenal fistula, and developed respiratory and hemodynamic failure associated also with a pulmonary embolism, requiring intensive care treatment corresponding to Clavien IV. No postoperative mortality was associated with duodenal stump leak. 

Six patients (2%) developed severe respiratory complications. Two with pneumonia required mechanical ventilation, and one was treated with a thoracocentesis due to parapneumonic pleural effusion. Thoracocentesis was also performed in two pleural effusions, and one patient required bi-level positive airway pressure (BiPAP) because of pulmonary atelectasis.

In the univariate analyses, EGJ tumor location (p=0.02), T3/T4 status (p=0.022), and multi-organ resection (p=0.05) were predictive factors for severe morbidity (Table 3). In multivariate analysis, EGJ tumor location (OR 3.3, 95% CI: 1.016-11.081, p=0.047) and T3/T4 involvement (OR 3.2, 95% CI: 1.056-9.707, p=0.04) remained statistically significant variables (Table 4).

**TABLE 3:** Univariable analysis of predictive factors of severe morbidity after gastrectomy

Variable	Severe morbidity	Mild or no morbidity	p
	n (%)	n (%)	
Age			
≥ 65	22 (14.5)	131 (85.6)	.087
< 65	10 (7.4)	126 (92.6)	
Gender			
Male	21 (10.8)	174 (89.2)	.813
Female	11 (11.7)	83 (88.3)	
Comorbidity			
Yes	24 (10.2)	211 (89.8)	.275
None	8 (17)	39 (83)	
ASA score			
I	6 (6.8)	82 (93.2)	.239
II	22 (13.7)	139 (83.3)	
III	3 (9.1)	30 (90.9)	
Smoking			
Yes	6 (9.7)	56 (90.3)	.808
No	26 (11.8)	194 (88.2)	
Alcohol consumption			
Yes	3 (16.3)	15 (83.3)	.441
No	29 (11)	25 (89)	
Hematocrit			
< 30%	5 (15.6)	27 (84.4)	.363
≥ 30%	25 (10.3)	220 (89.8)	
Body mass index			
< 18.5	2 (15.4)	11 (84.6)	.845
18.5 - 24.9	19 (12.1)	138 (87.9)	
25 - 29.9	8 (9.3)	78 (90.7)	
≥ 30	3 (9.1)	30 (90.9)	
Albumin (gr/dl)			
< 3.0	2 (14.3)	12 (85.7)	.650
≥ 3.0	28 (10.4)	241 (89.6)	
Tumor location			
EGJ	10 (23.8)	32 (76,2)	.02
Stomach upper third	11 (12.4)	78 (87.6)	
Stomach middle third	5 (6.6)	71 (93.4)	
Stomach lower third	6 (7.3)	76 (92.6)	
Neoadjuvant treatment			
No	32 (11.4)	249 (88.6)	.604
Chemotherapy	0 (0)	8 (100)	
Gastrectomy			
Open	30 (13)	201 (87)	.06
Laparoscopic	2 (3.4)	56 (96.6)	
Gastrectomy			
Total	25 (12.8)	171 (87.2)	.262
Subtotal	7 (7.5)	86 (92.5)	
Duodenal closure			
Hand-Sewn	17 (10.2)	149 (89.8)	.834
Mechanical	15 (12.2)	108 (87.8)	
Multiorgan resection			
Yes	12 (37.5)	57 (82.6)	.05
No	20 (9.1)	200 (90.9)	
Lymph node dissection			
D1	5 (12.2)	36 (87.8)	.786
D2	26 (10.6)	219 (89.4)	
Reconstruction route			
Retrocolic	20 (10.9)	163 (89.1)	.774
Antecolic	6 (8.7)	63 (91.3)	
T status			
T1 - T2	4 (4.3)	88 (95.7)	.022
T3 - T4	28 (14.2)	169 (85.8)	
N status			
N (+)	25 (13.7)	157 (83.3)	.091
N (-)	7 (6.5)	100 (93.5)	
Resection margin			
R0	28 (11.2)	221 (88.8)	1.
R1-2	4 (10)	36 (90)	

**TABLE 4:** Multivariable analysis of predictive factors of severe morbidity after gastrectomy

Variable	OR (CI 95%)	p
Tumor location		
Stomach lower third		
Stomach middle third	.8 (.252 - 3.006)	
Stomach upper third	1.7 (.587 - 5.105)	
Esophagogastric junction	3.3 (1.016 - 11.081)	.047
Multiorgan resection	1 (.412 - 2.485)	.980
T status		
T1 - T2		
T3 - T4	3.2 (1.056 - 9.707)	.04

**FIGURE 2:**
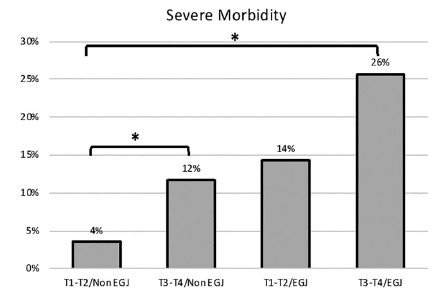
Risk of severe postoperative morbidity after gastrectomy according to T status and tumor location.

The frequency of severe complications was significantly higher for patients with a T3/T4 EGJ cancer, reaching 26% compared to the group of non-EGJ cases, and a T1/T2 status, with only 4% of severe complications (Figure 2). 

RR (Relative Risk) statistically significant with respect to T1-T2/Non EGJ group

In the T3/T4/EGJ group (n=38), the main severe complications were represented by two cases of severe esophagojejunal anastomosis leak (5%), two severe respiratory complications (5%), and three severe cardiac (8%) complications. Morbidity among EGJ cancer types according to the Siewert classification did not have a statistically significant difference (36% Siewert II and 18% Siewert III, p=0.37).

The groups T3/T4/non EGJ (RR 7.29; 95 % CI 2.096-25.32) and T3/T4/EGJ (RR 3.3; 95 % CI 1.012-10.91) presented statistically significant difference.

## DISCUSSION

Most studies describe and evaluate total postoperative morbidity^3^ ^,^ ^22^ ^,^ ^23^, and only more recent studies have evaluated postoperative morbidity according to complication severity^13^ ^,^ ^18^. Using the Clavien-Dindo classification, severe morbidity was present in 11% of patients after gastrectomy. We identified tumor location in the EGJ and gastric wall involvement beyond the muscular layer as predictors of severe postoperative morbidity.

The use of severity grading to evaluate postoperative complications offers several advantages. The Clavien system is easy to apply and has gained widespread use. The data collected from Clavien ≥3 cases allow us to focus on the complications with greater clinical significance and potentially life-threatening consequences and enables a more precise comparison between studies. Because levels I and II complications are often not fully documented across different centers, this is supported by the great variation of total morbidity description^3^ ^,^ ^13^ ^,^ ^18^ ^,^ ^22^ ^,^ ^23^, but a generally stable rate of severe complications between 9% and 12%^13^ ^,^ ^18^, was very similar to the rate described in this study (11%).

Previously reported risk factors for morbidity, such as age, preoperative co-morbidity, open surgical approach, multi-organ resection, splenectomy, or total gastrectomy observed in other series^3^ ^,^ ^11^ ^,^ ^12^ ^,^ ^13^ ^,^ ^14^ ^,^ ^18^ ^,^ ^22^ ^,^ ^23^ ^,^ ^26^, were not associated with severe morbidity in our study, probably because these factors were more often associated with overall morbidity. Laparoscopic approach was not associated with a significantly lower rate of severe morbidity. This result may be due in part to the less frequent use of laparoscopic gastrectomy in our study, particularly in the EGJ location.

EGJ cancer is more prevalent in Western centers and represents a subgroup of esophagogastric malignancies that have special staging and treatment modalities, depending on patients’ and tumors’ factors and mainly tumor location as described by Siewert classification^10,27,^. In our study, we included Siewert types II and III tumors treated with gastrectomy, most of them with transhiatal distal esophagectomy and mediastinal esophagojejunal anastomosis. In the group of patients with EGJ location and a T3/T4 status, significantly high severe morbidity reached 26%, associated with a higher rate of esophagojejunal anastomosis leak and cardiorespiratory complications. This might have been due to the technical difficulties in the resection phase of the operation because of the manipulation of the pericardium and both pleurae. Also the technical difficulties to perform an esophagojejunal anastomosis high in the mediastinum with restricted space or the peripheral inflammation associated with an EGJ and T3/T4 cancer may have facilitated second injuries, future bleeding, or leaks that eventually explained their risk association.

Generally, gastrectomy is viewed as a procedure with lower morbidity compared to esophagectomy^7^, but our data show that for T3/T4 and EGJ tumors this may not be the case. These morbidity data must be considered in the preoperative planning in patients who may be candidates to either esophagectomy or gastrectomy, according to local morbidity and mortality results from each procedure^4^. In patients with a higher risk of severe complications, the preoperative nutritional and physical condition in the weeks previous to surgery needs to be improved, the postoperative management should be optimized, and these conditions carefully monitored for possible complications.

Several studies of esophageal and colon cancers have shown that postoperative complications, by themselves, are associated with worse oncological survival^1,30,^. For gastric cancer, the data are contradictory on how postoperative complications affect long-term survival^6,16,28,^. The follow-up of our patients will allow us to define whether global, severe or infectious complications change survival.

This study has a limitation that corresponds to a retrospective cohort, and some factors, such as preoperative weight loss, intraoperative bleeding, pre- or intraoperative red blood cell transfusion, were not available to include in the analysis.

## CONCLUSION

Eleven percent of patients present severe morbidity after gastrectomy. EGJ tumor location and gastric wall involvement beyond the muscular layer represent predictors of severe postoperative morbidity. This risk stratification allows a more precise decision-making process for patient selection, evaluation, and optimization as well as improved counseling about the risks of surgery. 
